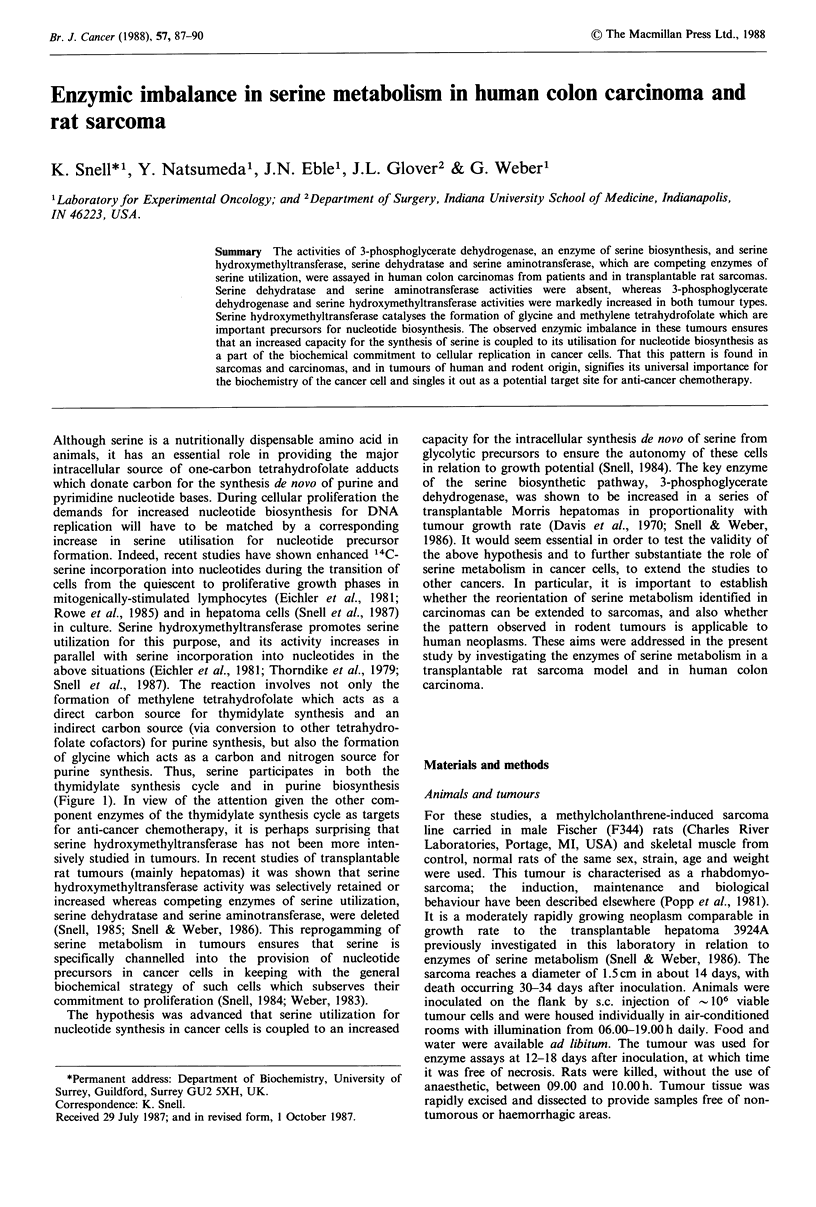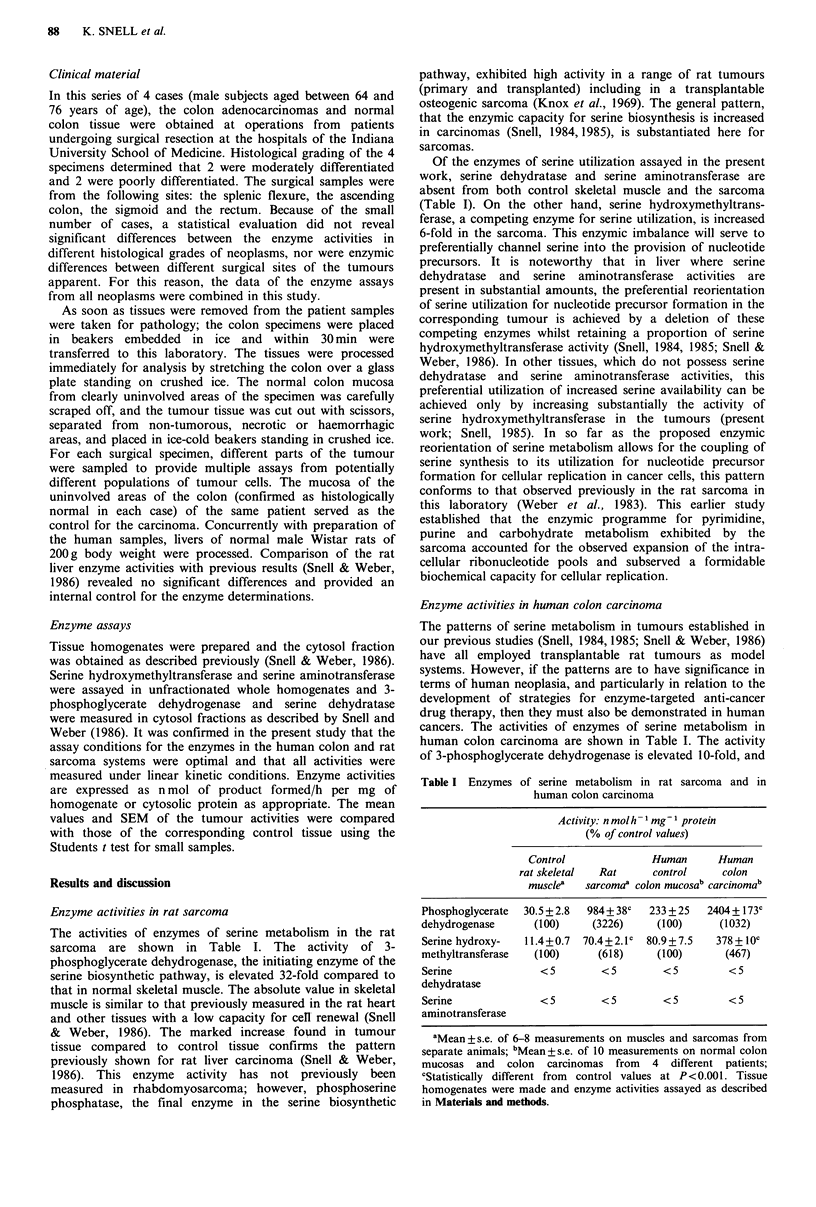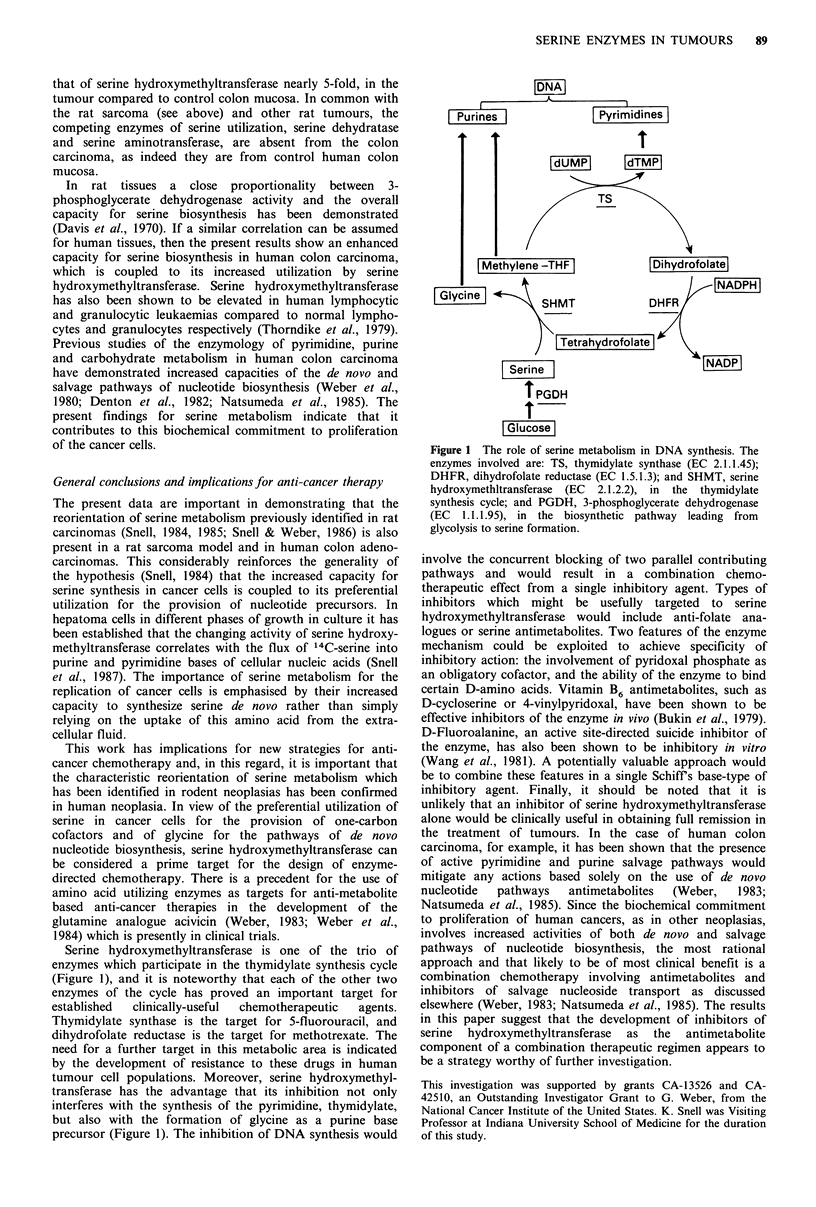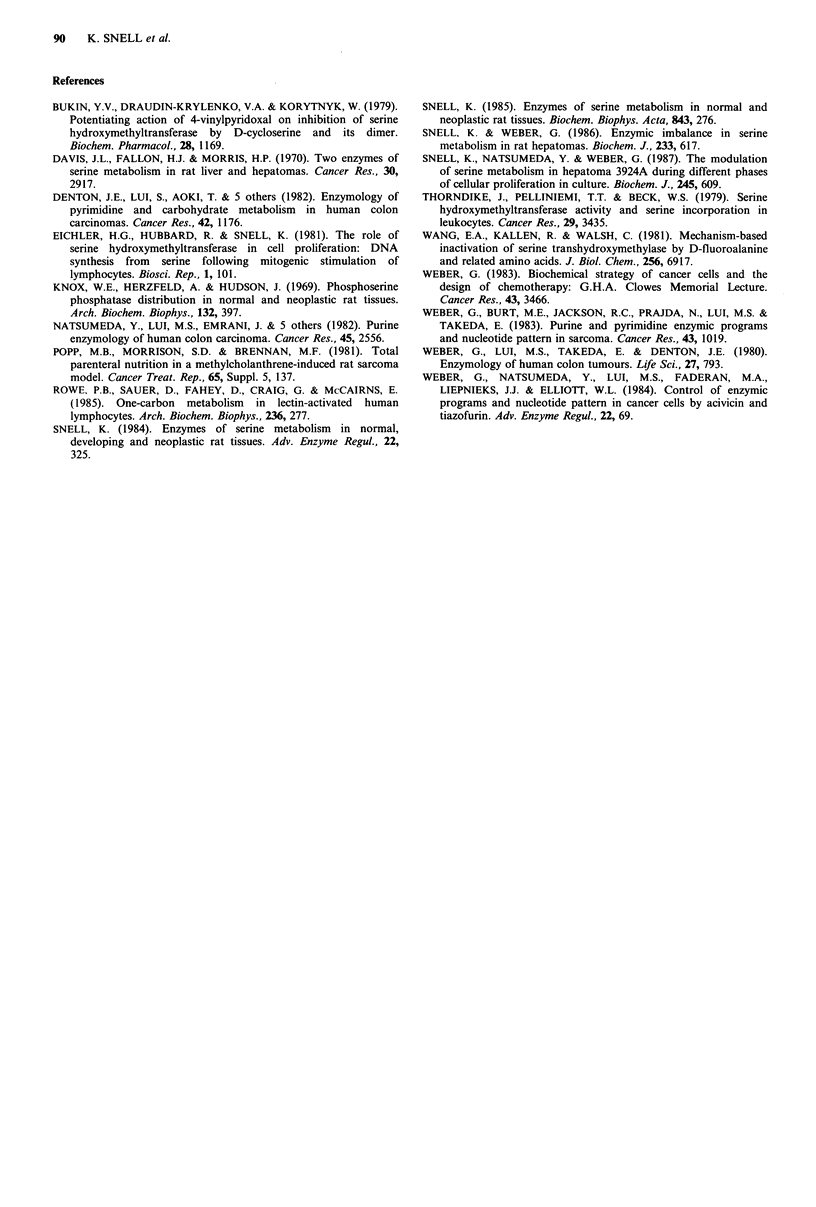# Enzymic imbalance in serine metabolism in human colon carcinoma and rat sarcoma.

**DOI:** 10.1038/bjc.1988.15

**Published:** 1988-01

**Authors:** K. Snell, Y. Natsumeda, J. N. Eble, J. L. Glover, G. Weber

**Affiliations:** Laboratory for Experimental Oncology, Indiana University School of Medicine, Indianapolis 46223.

## Abstract

The activities of 3-phosphoglycerate dehydrogenase, an enzyme of serine biosynthesis, and serine hydroxymethyltransferase, serine dehydratase and serine aminotransferase, which are competing enzymes of serine utilization, were assayed in human colon carcinomas from patients and in transplantable rat sarcomas. Serine dehydratase and serine aminotransferase activities were absent, whereas 3-phosphoglycerate dehydrogenase and serine hydroxymethyltransferase activities were markedly increased in both tumour types. Serine hydroxymethyltransferase catalyses the formation of glycine and methylene tetrahydrofolate which are important precursors for nucleotide biosynthesis. The observed enzymic imbalance in these tumours ensures that an increased capacity for the synthesis of serine is coupled to its utilisation for nucleotide biosynthesis as a part of the biochemical commitment to cellular replication in cancer cells. That this pattern is found in sarcomas and carcinomas, and in tumours of human and rodent origin, signifies its universal importance for the biochemistry of the cancer cell and singles it out as a potential target site for anti-cancer chemotherapy.


Br. J. Cancer (1988), 57, 87–90 © The Macmillan Press Ltd., 1988

### Enzymic imbalance in serine metabolism in human colon carcinoma and rat sarcoma

K. Snell*[1], Y. Natsumeda[1], J.N. Eble[1], J.L. Glover[2] & G. Weber[1]

[1] *Laboratory for Experimental Oncology; and* [2] *Department of Surgery, Indiana University School of Medicine, Indianapolis, IN 46223, USA.*

**Summary** The activities of 3-phosphoglycerate dehydrogenase, an enzyme of serine biosynthesis, and serine hydroxymethyltransferase, serine dehydratase and serine aminotransferase, which are competing enzymes of serine utilization, were assayed in human colon carcinomas from patients and in transplantable rat sarcomas. Serine dehydratase and serine aminotransferase activities were absent, whereas 3-phosphoglycerate dehydrogenase and serine hydroxymethyltransferase activities were markedly increased in both tumour types. Serine hydroxymethyltransferase catalyses the formation of glycine and methylene tetrahydrofolate which are important precursors for nucleotide biosynthesis. The observed enzymic imbalance in these tumours ensures that an increased capacity for the synthesis of serine is coupled to its utilisation for nucleotide biosynthesis as a part of the biochemical commitment to cellular replication in cancer cells. That this pattern is found in sarcomas and carcinomas, and in tumours of human and rodent origin, signifies its universal importance for the biochemistry of the cancer cell and singles it out as a potential target site for anti-cancer chemotherapy.

Although serine is a nutritionally dispensable amino acid in animals, it has an essential role in providing the major intracellular source of one-carbon tetrahydrofolate adducts which donate carbon for the synthesis *de novo* of purine and pyrimidine nucleotide bases. During cellular proliferation the demands for increased nucleotide biosynthesis for DNA replication will have to be matched by a corresponding increase in serine utilisation for nucleotide precursor formation. Indeed, recent studies have shown enhanced $^{14}C$-serine incorporation into nucleotides during the transition of cells from the quiescent to proliferative growth phases in mitogenically-stimulated lymphocytes (Eichler *et al.*, 1981; Rowe *et al.*, 1985) and in hepatoma cells (Snell *et al.*, 1987) in culture. Serine hydroxymethyltransferase promotes serine utilization for this purpose, and its activity increases in parallel with serine incorporation into nucleotides in the above situations (Eichler *et al.*, 1981; Thorndike *et al.*, 1979; Snell *et al.*, 1987). The reaction involves not only the formation of methylene tetrahydrofolate which acts as a direct carbon source for thymidylate synthesis and an indirect carbon source (via conversion to other tetrahydrofolate cofactors) for purine synthesis, but also the formation of glycine which acts as a carbon and nitrogen source for purine synthesis. Thus, serine participates in both the thymidylate synthesis cycle and in purine biosynthesis (Figure 1). In view of the attention given the other component enzymes of the thymidylate synthesis cycle as targets for anti-cancer chemotherapy, it is perhaps surprising that serine hydroxymethyltransferase has not been more intensively studied in tumours. In recent studies of transplantable rat tumours (mainly hepatomas) it was shown that serine hydroxymethyltransferase activity was selectively retained or increased whereas competing enzymes of serine utilization, serine dehydratase and serine aminotransferase, were deleted (Snell, 1985; Snell & Weber, 1986). This reprogamming of serine metabolism in tumours ensures that serine is specifically channelled into the provision of nucleotide precursors in cancer cells in keeping with the general biochemical strategy of such cells which subserves their commitment to proliferation (Snell, 1984; Weber, 1983).

The hypothesis was advanced that serine utilization for nucleotide synthesis in cancer cells is coupled to an increased capacity for the intracellular synthesis *de novo* of serine from glycolytic precursors to ensure the autonomy of these cells in relation to growth potential (Snell, 1984). The key enzyme of the serine biosynthetic pathway, 3-phosphoglycerate dehydrogenase, was shown to be increased in a series of transplantable Morris hepatomas in proportionality with tumour growth rate (Davis *et al.*, 1970; Snell & Weber, 1986). It would seem essential in order to test the validity of the above hypothesis and to further substantiate the role of serine metabolism in cancer cells, to extend the studies to other cancers. In particular, it is important to establish whether the reorientation of serine metabolism identified in carcinomas can be extended to sarcomas, and also whether the pattern observed in rodent tumours is applicable to human neoplasms. These aims were addressed in the present study by investigating the enzymes of serine metabolism in a transplantable rat sarcoma model and in human colon carcinoma.

### Materials and methods

### *Animals and tumours*

For these studies, a methylcholanthrene-induced sarcoma line carried in male Fischer (F344) rats (Charles River Laboratories, Portage, MI, USA) and skeletal muscle from control, normal rats of the same sex, strain, age and weight were used. This tumour is characterised as a rhabdomyosarcoma; the induction, maintenance and biological behaviour have been described elsewhere (Popp *et al.*, 1981). It is a moderately rapidly growing neoplasm comparable in growth rate to the transplantable hepatoma 3924A previously investigated in this laboratory in relation to enzymes of serine metabolism (Snell & Weber, 1986). The sarcoma reaches a diameter of 1.5 cm in about 14 days, with death occurring 30–34 days after inoculation. Animals were inoculated on the flank by s.c. injection of $\sim 10^6$ viable tumour cells and were housed individually in air-conditioned rooms with illumination from 06.00–19.00 h daily. Food and water were available *ad libitum*. The tumour was used for enzyme assays at 12–18 days after inoculation, at which time it was free of necrosis. Rats were killed, without the use of anaesthetic, between 09.00 and 10.00 h. Tumour tissue was rapidly excised and dissected to provide samples free of non-tumorous or haemorrhagic areas.

*Permanent address: Department of Biochemistry, University of Surrey, Guildford, Surrey GU2 5XH, UK.
Correspondence: K. Snell.
Received 29 July 1987; and in revised form, 1 October 1987.

*Clinical material*

In this series of 4 cases (male subjects aged between 64 and 76 years of age), the colon adenocarcinomas and normal colon tissue were obtained at operations from patients undergoing surgical resection at the hospitals of the Indiana University School of Medicine. Histological grading of the 4 specimens determined that 2 were moderately differentiated and 2 were poorly differentiated. The surgical samples were from the following sites: the splenic flexure, the ascending colon, the sigmoid and the rectum. Because of the small number of cases, a statistical evaluation did not reveal significant differences between the enzyme activities in different histological grades of neoplasms, nor were enzymic differences between different surgical sites of the tumours apparent. For this reason, the data of the enzyme assays from all neoplasms were combined in this study.

As soon as tissues were removed from the patient samples were taken for pathology; the colon specimens were placed in beakers embedded in ice and within 30 min were transferred to this laboratory. The tissues were processed immediately for analysis by stretching the colon over a glass plate standing on crushed ice. The normal colon mucosa from clearly uninvolved areas of the specimen was carefully scraped off, and the tumour tissue was cut out with scissors, separated from non-tumorous, necrotic or haemorrhagic areas, and placed in ice-cold beakers standing in crushed ice. For each surgical specimen, different parts of the tumour were sampled to provide multiple assays from potentially different populations of tumour cells. The mucosa of the uninvolved areas of the colon (confirmed as histologically normal in each case) of the same patient served as the control for the carcinoma. Concurrently with preparation of the human samples, livers of normal male Wistar rats of 200 g body weight were processed. Comparison of the rat liver enzyme activities with previous results (Snell & Weber, 1986) revealed no significant differences and provided an internal control for the enzyme determinations.

*Enzyme assays*

Tissue homogenates were prepared and the cytosol fraction was obtained as described previously (Snell & Weber, 1986). Serine hydroxymethyltransferase and serine aminotransferase were assayed in unfractionated whole homogenates and 3-phosphoglycerate dehydrogenase and serine dehydratase were measured in cytosol fractions as described by Snell and Weber (1986). It was confirmed in the present study that the assay conditions for the enzymes in the human colon and rat sarcoma systems were optimal and that all activities were measured under linear kinetic conditions. Enzyme activities are expressed as n mol of product formed/h per mg of homogenate or cytosolic protein as appropriate. The mean values and SEM of the tumour activities were compared with those of the corresponding control tissue using the Students $t$ test for small samples.

### Results and discussion

*Enzyme activities in rat sarcoma*

The activities of enzymes of serine metabolism in the rat sarcoma are shown in Table I. The activity of 3-phosphoglycerate dehydrogenase, the initiating enzyme of the serine biosynthetic pathway, is elevated 32-fold compared to that in normal skeletal muscle. The absolute value in skeletal muscle is similar to that previously measured in the rat heart and other tissues with a low capacity for cell renewal (Snell & Weber, 1986). The marked increase found in tumour tissue compared to control tissue confirms the pattern previously shown for rat liver carcinoma (Snell & Weber, 1986). This enzyme activity has not previously been measured in rhabdomyosarcoma; however, phosphoserine phosphatase, the final enzyme in the serine biosynthetic pathway, exhibited high activity in a range of rat tumours (primary and transplanted) including in a transplantable osteogenic sarcoma (Knox et al., 1969). The general pattern, that the enzymic capacity for serine biosynthesis is increased in carcinomas (Snell, 1984, 1985), is substantiated here for sarcomas.

Of the enzymes of serine utilization assayed in the present work, serine dehydratase and serine aminotransferase are absent from both control skeletal muscle and the sarcoma (Table I). On the other hand, serine hydroxymethyltransferase, a competing enzyme for serine utilization, is increased 6-fold in the sarcoma. This enzymic imbalance will serve to preferentially channel serine into the provision of nucleotide precursors. It is noteworthy that in liver where serine dehydratase and serine aminotransferase activities are present in substantial amounts, the preferential reorientation of serine utilization for nucleotide precursor formation in the corresponding tumour is achieved by a deletion of these competing enzymes whilst retaining a proportion of serine hydroxymethyltransferase activity (Snell, 1984, 1985; Snell & Weber, 1986). In other tissues, which do not possess serine dehydratase and serine aminotransferase activities, this preferential utilization of increased serine availability can be achieved only by increasing substantially the activity of serine hydroxymethyltransferase in the tumours (present work; Snell, 1985). In so far as the proposed enzymic reorientation of serine metabolism allows for the coupling of serine synthesis to its utilization for nucleotide precursor formation for cellular replication in cancer cells, this pattern conforms to that observed previously in the rat sarcoma in this laboratory (Weber et al., 1983). This earlier study established that the enzymic programme for pyrimidine, purine and carbohydrate metabolism exhibited by the sarcoma accounted for the observed expansion of the intracellular ribonucleotide pools and subserved a formidable biochemical capacity for cellular replication.

*Enzyme activities in human colon carcinoma*

The patterns of serine metabolism in tumours established in our previous studies (Snell, 1984, 1985; Snell & Weber, 1986) have all employed transplantable rat tumours as model systems. However, if the patterns are to have significance in terms of human neoplasia, and particularly in relation to the development of strategies for enzyme-targeted anti-cancer drug therapy, then they must also be demonstrated in human cancers. The activities of enzymes of serine metabolism in human colon carcinoma are shown in Table I. The activity of 3-phosphoglycerate dehydrogenase is elevated 10-fold, and

**Table I** Enzymes of serine metabolism in rat sarcoma and in human colon carcinoma

| | Activity: $n mol\,h^{-1}\,mg^{-1}$ protein (% of control values) | | | |
|---|---|---|---|---|
| | Control rat skeletal muscle[a] | Rat sarcoma[a] | Human control colon mucosa[b] | Human colon carcinoma[b] |
| Phosphoglycerate dehydrogenase | $30.5 \pm 2.8$ (100) | $984 \pm 38^{c}$ (3226) | $233 \pm 25$ (100) | $2404 \pm 173^{c}$ (1032) |
| Serine hydroxy-methyltransferase | $11.4 \pm 0.7$ (100) | $70.4 \pm 2.1^{c}$ (618) | $80.9 \pm 7.5$ (100) | $378 \pm 10^{c}$ (467) |
| Serine dehydratase | <5 | <5 | <5 | <5 |
| Serine aminotransferase | <5 | <5 | <5 | <5 |

[a]Mean ± s.e. of 6–8 measurements on muscles and sarcomas from separate animals; [b]Mean ± s.e. of 10 measurements on normal colon mucosas and colon carcinomas from 4 different patients; [c]Statistically different from control values at $P<0.001$. Tissue homogenates were made and enzyme activities assayed as described in **Materials and methods**.

that of serine hydroxymethyltransferase nearly 5-fold, in the tumour compared to control colon mucosa. In common with the rat sarcoma (see above) and other rat tumours, the competing enzymes of serine utilization, serine dehydratase and serine aminotransferase, are absent from the colon carcinoma, as indeed they are from control human colon mucosa.

In rat tissues a close proportionality between 3-phosphoglycerate dehydrogenase activity and the overall capacity for serine biosynthesis has been demonstrated (Davis *et al.*, 1970). If a similar correlation can be assumed for human tissues, then the present results show an enhanced capacity for serine biosynthesis in human colon carcinoma, which is coupled to its increased utilization by serine hydroxymethyltransferase. Serine hydroxymethyltransferase has also been shown to be elevated in human lymphocytic and granulocytic leukaemias compared to normal lymphocytes and granulocytes respectively (Thorndike *et al.*, 1979). Previous studies of the enzymology of pyrimidine, purine and carbohydrate metabolism in human colon carcinoma have demonstrated increased capacities of the *de novo* and salvage pathways of nucleotide biosynthesis (Weber *et al.*, 1980; Denton *et al.*, 1982; Natsumeda *et al.*, 1985). The present findings for serine metabolism indicate that it contributes to this biochemical commitment to proliferation of the cancer cells.

### *General conclusions and implications for anti-cancer therapy*

The present data are important in demonstrating that the reorientation of serine metabolism previously identified in rat carcinomas (Snell, 1984, 1985; Snell & Weber, 1986) is also present in a rat sarcoma model and in human colon adenocarcinomas. This considerably reinforces the generality of the hypothesis (Snell, 1984) that the increased capacity for serine synthesis in cancer cells is coupled to its preferential utilization for the provision of nucleotide precursors. In hepatoma cells in different phases of growth in culture it has been established that the changing activity of serine hydroxymethyltransferase correlates with the flux of $^{14}C$-serine into purine and pyrimidine bases of cellular nucleic acids (Snell *et al.*, 1987). The importance of serine metabolism for the replication of cancer cells is emphasised by their increased capacity to synthesize serine *de novo* rather than simply relying on the uptake of this amino acid from the extracellular fluid.

This work has implications for new strategies for anti-cancer chemotherapy and, in this regard, it is important that the characteristic reorientation of serine metabolism which has been identified in rodent neoplasias has been confirmed in human neoplasia. In view of the preferential utilization of serine in cancer cells for the provision of one-carbon cofactors and of glycine for the pathways of *de novo* nucleotide biosynthesis, serine hydroxymethyltransferase can be considered a prime target for the design of enzyme-directed chemotherapy. There is a precedent for the use of amino acid utilizing enzymes as targets for anti-metabolite based anti-cancer therapies in the development of the glutamine analogue acivicin (Weber, 1983; Weber *et al.*, 1984) which is presently in clinical trials.

Serine hydroxymethyltransferase is one of the trio of enzymes which participate in the thymidylate synthesis cycle (Figure 1), and it is noteworthy that each of the other two enzymes of the cycle has proved an important target for established clinically-useful chemotherapeutic agents. Thymidylate synthase is the target for 5-fluorouracil, and dihydrofolate reductase is the target for methotrexate. The need for a further target in this metabolic area is indicated by the development of resistance to these drugs in human tumour cell populations. Moreover, serine hydroxymethyltransferase has the advantage that its inhibition not only interferes with the synthesis of the pyrimidine, thymidylate, but also with the formation of glycine as a purine base precursor (Figure 1). The inhibition of DNA synthesis would involve the concurrent blocking of two parallel contributing pathways and would result in a combination chemotherapeutic effect from a single inhibitory agent. Types of inhibitors which might be usefully targeted to serine hydroxymethyltransferase would include anti-folate analogues or serine antimetabolites. Two features of the enzyme mechanism could be exploited to achieve specificity of inhibitory action: the involvement of pyridoxal phosphate as an obligatory cofactor, and the ability of the enzyme to bind certain D-amino acids. Vitamin $B_6$ antimetabolites, such as D-cycloserine or 4-vinylpyridoxal, have been shown to be effective inhibitors of the enzyme *in vivo* (Bukin *et al.*, 1979). D-Fluoroalanine, an active site-directed suicide inhibitor of the enzyme, has also been shown to be inhibitory *in vitro* (Wang *et al.*, 1981). A potentially valuable approach would be to combine these features in a single Schiff's base-type of inhibitory agent. Finally, it should be noted that it is unlikely that an inhibitor of serine hydroxymethyltransferase alone would be clinically useful in obtaining full remission in the treatment of tumours. In the case of human colon carcinoma, for example, it has been shown that the presence of active pyrimidine and purine salvage pathways would mitigate any actions based solely on the use of *de novo* nucleotide pathways antimetabolites (Weber, 1983; Natsumeda *et al.*, 1985). Since the biochemical commitment to proliferation of human cancers, as in other neoplasias, involves increased activities of both *de novo* and salvage pathways of nucleotide biosynthesis, the most rational approach and that likely to be of most clinical benefit is a combination chemotherapy involving antimetabolites and inhibitors of salvage nucleoside transport as discussed elsewhere (Weber, 1983; Natsumeda *et al.*, 1985). The results in this paper suggest that the development of inhibitors of serine hydroxymethyltransferase as the antimetabolite component of a combination therapeutic regimen appears to be a strategy worthy of further investigation.

**Figure 1** The role of serine metabolism in DNA synthesis. The enzymes involved are: TS, thymidylate synthase (EC 2.1.1.45); DHFR, dihydrofolate reductase (EC 1.5.1.3); and SHMT, serine hydroxymethltransferase (EC 2.1.2.2), in the thymidylate synthesis cycle; and PGDH, 3-phosphoglycerate dehydrogenase (EC 1.1.1.95), in the biosynthetic pathway leading from glycolysis to serine formation.

This investigation was supported by grants CA-13526 and CA-42510, an Outstanding Investigator Grant to G. Weber, from the National Cancer Institute of the United States. K. Snell was Visiting Professor at Indiana University School of Medicine for the duration of this study.

### References

BUKIN, Y.V., DRAUDIN-KRYLENKO, V.A. & KORYTNYK, W. (1979). Potentiating action of 4-vinylpyridoxal on inhibition of serine hydroxymethyltransferase by D-cycloserine and its dimer. *Biochem. Pharmacol.*, **28**, 1169.

DAVIS, J.L., FALLON, H.J. & MORRIS, H.P. (1970). Two enzymes of serine metabolism in rat liver and hepatomas. *Cancer Res.*, **30**, 2917.

DENTON, J.E., LUI, S., AOKI, T. & 5 others (1982). Enzymology of pyrimidine and carbohydrate metabolism in human colon carcinomas. *Cancer Res.*, **42**, 1176.

EICHLER, H.G., HUBBARD, R. & SNELL, K. (1981). The role of serine hydroxymethyltransferase in cell proliferation: DNA synthesis from serine following mitogenic stimulation of lymphocytes. *Biosci. Rep.*, **1**, 101.

KNOX, W.E., HERZFELD, A. & HUDSON, J. (1969). Phosphoserine phosphatase distribution in normal and neoplastic rat tissues. *Arch. Biochem. Biophys.*, **132**, 397.

NATSUMEDA, Y., LUI, M.S., EMRANI, J. & 5 others (1982). Purine enzymology of human colon carcinoma. *Cancer Res.*, **45**, 2556.

POPP, M.B., MORRISON, S.D. & BRENNAN, M.F. (1981). Total parenteral nutrition in a methylcholanthrene-induced rat sarcoma model. *Cancer Treat. Rep.*, **65**, Suppl. 5, 137.

ROWE, P.B., SAUER, D., FAHEY, D., CRAIG, G. & MCCAIRNS, E. (1985). One-carbon metabolism in lectin-activated human lymphocytes. *Arch. Biochem. Biophys.*, **236**, 277.

SNELL, K. (1984). Enzymes of serine metabolism in normal, developing and neoplastic rat tissues. *Adv. Enzyme Regul.*, **22**, 325.

SNELL, K. (1985). Enzymes of serine metabolism in normal and neoplastic rat tissues. *Biochem. Biophys. Acta*, **843**, 276.

SNELL, K. & WEBER, G. (1986). Enzymic imbalance in serine metabolism in rat hepatomas. *Biochem. J.*, **233**, 617.

SNELL, K., NATSUMEDA, Y. & WEBER, G. (1987). The modulation of serine metabolism in hepatoma 3924A during different phases of cellular proliferation in culture. *Biochem. J.*, **245**, 609.

THORNDIKE, J., PELLINIEMI, T.T. & BECK, W.S. (1979). Serine hydroxymethyltransferase activity and serine incorporation in leukocytes. *Cancer Res.*, **29**, 3435.

WANG, E.A., KALLEN, R. & WALSH, C. (1981). Mechanism-based inactivation of serine transhydroxymethylase by D-fluoroalanine and related amino acids. *J. Biol. Chem.*, **256**, 6917.

WEBER, G. (1983). Biochemical strategy of cancer cells and the design of chemotherapy: G.H.A. Clowes Memorial Lecture. *Cancer Res.*, **43**, 3466.

WEBER, G., BURT, M.E., JACKSON, R.C., PRAJDA, N., LUI, M.S. & TAKEDA, E. (1983). Purine and pyrimidine enzymic programs and nucleotide pattern in sarcoma. *Cancer Res.*, **43**, 1019.

WEBER, G., LUI, M.S., TAKEDA, E. & DENTON, J.E. (1980). Enzymology of human colon tumours. *Life Sci.*, **27**, 793.

WEBER, G., NATSUMEDA, Y., LUI, M.S., FADERAN, M.A., LIEPNIEKS, J.J. & ELLIOTT, W.L. (1984). Control of enzymic programs and nucleotide pattern in cancer cells by acivicin and tiazofurin. *Adv. Enzyme Regul.*, **22**, 69.